# ACROPath Oligometastases: The American College of Radiation Oncology Clinical Pathway

**DOI:** 10.7759/cureus.74098

**Published:** 2024-11-20

**Authors:** Christopher D Jahraus, Paul E Wallner, Dwight E Heron, William Crook, Steven E Finkelstein, Alexander A Harris, Larry Kestin, Evan Landau, Douglas Rivera, Tarita O Thomas, Bridget F Koontz

**Affiliations:** 1 Radiation Oncology, ACROPath Project, American College of Radiation Oncology, Alabaster, USA; 2 Radiation Oncology, Generations Radiotherapy & Oncology PC, Alabaster, USA; 3 Radiation Oncology, American College of Radiation Oncology, Moorestown, USA; 4 Radiation Oncology, Mercy Health, Youngstown, USA; 5 Radiation Oncology, American College of Radiation Oncology, Youngstown, USA; 6 Radiaiton Oncology, GenesisCare, Fort Pierce, USA; 7 Radiation Oncology, Associated Medical Professionals of New York, Syracuse, USA; 8 Radiation Oncology, Radiation Oncology Consultants, Chicago, USA; 9 Radiation Oncology, Michigan Healthcare Professionals (MHP) Radiation Oncology Institute, Farmington Hills, USA; 10 Radiation Oncology, GenesisCare, Fort Lauderdale, USA; 11 Radiation Oncology, Austin Cyberknife, Austin, USA; 12 Radiation Oncology, Central Texas Cancer Centers, Georgetown, USA; 13 Radiation Oncology, Feinberg School of Medicine, Northwestern University, Chicago, USA; 14 Radiation Oncology, AdventHealth, Orlando, USA

**Keywords:** cancer, clinical pathway, decision support tools, oligometastases, radiotherapy

## Abstract

Radiation oncology is among the most data-driven specialties in medicine. Recently, a wealth of peer-reviewed data has been published supporting the treatment of oligometastatic malignancies, demonstrating improved survival with metastasis-directed therapy, such as stereotactic body radiation therapy (SBRT), when combined with appropriate patient selection and treatment. However, there are currently few, if any, established guidelines that synthesize the abundance of data specific to radiotherapy into a single, easily accessed resource for clinicians. ACROPath® is a major initiative of the American College of Radiation Oncology (ACRO) that aims to present aggregated clinical pathway data in a highly usable format that is readily accessible to clinicians at the point of care in real time. The oligometastases pathway is the first published algorithm in this collection, with additional pathways anticipated in future publications. Clinical radiation oncologists with expertise in the treatment and management of oligometastatic disease were recruited from across ACRO’s diverse membership, including both academic and private practice physicians, to ensure a broad-based experience and insight. Individual participants were assigned subsections of the pathway for guideline development, and then, each subsection was presented to the full group for evaluation and consensus development based on published data. Rather than presenting an unstructured set of treatment options, as is common in other treatment guidelines, this initiative aimed to categorize appropriate treatments based on published clinical evidence in a hierarchy further ranked by efficacy, toxicity, and cost. Based on these strata, treatment recommendations were collated and grouped into three rank categories (gold, silver, or bronze) to denote the degree of applicability. The team assembled an interactive document that will eventually be available online, and it is summarized in detail here. Recommendations are grouped both by the anatomic site of metastasis and by the primary tumor type, recognizing that original histology might impact the treatment differently in different anatomic locations. After a review of available published clinical evidence, the committee reached a consensus on all recommendations presented, categorizing each option as gold, silver, or bronze to guide clinicians appropriately. This first iteration of ACROPath® Oligometastases represents one of the few comprehensive clinical decision support tools available for managing patients with limited metastatic disease. It presents available data in a highly accessible, easily used reference, which will be formally reviewed and updated by the committee as frequently as emerging data requires, likely at six- to 12-month intervals.

## Introduction

With the increasing complexity of cancer care and the evolution of treatment paradigms, physician decision support tools are becoming an increasingly important part of the radiation oncologists’ armamentarium. The abundance of data and data sources being generated and published is expanding exponentially, making it increasingly difficult for radiation oncologists to stay current with the growing body of emerging data across all cancer types. Moreover, the prior authorization process used by many insurers now requires citing appropriate references to support a given approach to care, particularly when the situation is uncommon. In 2022, the ACRO Board of Chancellors (BoC) developed a plan for assembling a broad set of practice support tools organized into clinical pathways based primarily on disease sites. Early in the process, the organizers realized oligometastatic disease represents a unique category of cancer presentation with management challenges that warrant its own pathway. This publication and the forthcoming online content represent the culmination of the effort and the first public release of an ACROPath® document.

Defining oligometastatic disease

The Hellman-Weichselbaum hypothesis originally defined oligometastasis as a transient phase in cancer progression between primary localized cancer and widespread polymetastatic dissemination [1]. Several numerical or anatomic criteria have been proposed to define oligometastatic disease [2,3], although recent and ongoing studies continue to vary in their specific definitions. Our panel acknowledged that five or fewer lesions involving three or fewer organs are generally accepted by most authors as potentially amenable to aggressive ablative management, with exceptions for cases involving more lesions if all can be definitively treated. We have considered that multiple brain metastases represent a single site of disease, provided all lesions can be treated with appropriate radiotherapy. The European Organisation for Research and Treatment of Cancer (EORTC) categorizes patients with limited metastases as being “genuine,” having no history of polymetastatic disease, and “induced,” having had prior polymetastatic disease which responded to systemic therapy [4]. The same group defined “oligoprogression,” meaning that a patient has progressive disease in a small number of areas. Our recommendations are designed to apply to patients in potentially all of these situations.

Value of treating oligometastases

Aggressive treatment of oligometastatic disease has proven to be one of the most substantial advances in radiation oncology over the past decade. Available scientific data has demonstrated improved outcomes, including survival for certain cancers. In patients with oligometastatic colorectal cancer, treatment of metastatic lesions with stereotactic body radiation therapy (SBRT) yielded a five-year overall survival (OS), local progression-free survival (PFS), and distant PFS of 45%, 83%, and 27%, respectively, with no significant change in patient-reported quality of life (QoL) [5]. One of the most often cited studies of SBRT’s utility in treating oligometastases, the SABR-COMET study, demonstrated that with a controlled primary tumor and 1-5 metastases, treatment of the individual metastatic sites resulted in an almost 50% improvement in OS, increasing from 28 months in the control group to 41 months in the SBRT-treated group [6]. A prospective phase 2 trial of 147 patients demonstrated efficacy with statistically improved patient-reported QoL scores at six and 12 months [7].

SBRT is a high-value treatment, with a total cost for American patients insured by Medicare typically under US$10,000 for an entire course, compared to the average wholesale price of a single 1500-mg dose of durvalumab that exceeds $13,000 and is typically administered every two weeks for an indeterminate period [8]. A cost-effectiveness analysis of SBRT for oligometastases found that SBRT compared favorably to standard systemic drug therapy, leading to an improvement of 1.88 quality-adjusted life years [9].

## Technical report

ACRO is a diverse medical specialty organization comprising domestic and international academic, private practice, clinicians, physicists, and administrators in radiation oncology. The scientific committee identified subcommittee leaders for each ACROPath® disease site, and each subcommittee chair worked with the program chairs to recruit appropriate topic-specific participants. Individuals from a range of practice settings were recruited to ensure a balance of diverse practice patterns and philosophies. Subcommittee chairs assigned participants to subsections of the pathway for preliminary guideline development. This process included a thorough review of the available literature to ensure the guidelines are evidence-based. Thereafter, each section was presented to the full group for editing.

All recommendations in the pathway are based on peer-reviewed evidence and rationale, and no portion of the pathway recommendations is considered “experimental” or otherwise lacking sufficient literature support for routine use. The committee is aware of other guidelines ranking the quality of data using specific metrics, such as grading individual studies and evidence, though we have intentionally avoided listing studies encyclopedically with a particular grade, as many of our constituents find this approach confusing, making the selection of a particular approach more difficult. We have therefore opted to rank recommended approaches as gold, silver, or bronze based on the literature. Gold recommendations are those that are typically appropriate for all patients, with limited exceptions, and are generally the preferred approach. Silver recommendations are usually regarded as equally efficacious as gold, but potentially not as convenient or potentially less well-supported in the literature than gold regimens. Bronze recommendations are those that represent a reasonable compromise when gold or silver recommendations cannot be achieved or when the patient’s situation necessitates special consideration. All recommendations presented in this or future ACROPath® documents are considered reasonable and appropriate standards of care (SOC), provided there is proper clinical rationale. Recognizing the diversity of practice situations, we acknowledge that there may be circumstances where the equipment and resources needed to administer gold-standard treatments are not readily available. In these circumstances, the clinician should weigh the merits of a silver or bronze approach against the feasibility of obtaining care for the patient at a better-equipped center. Only the treating physician and the patient can collaboratively make the optimal choice, and the accessibility of care must be given appropriate weight in the decision-making process.

As with all guidelines and clinical support tools, ACROPath® recommendations should never replace the intellect, logic, and reasoning of an experienced clinician, in combination with patient and caregiver dialogue and shared decision-making. Furthermore, failure to follow this decision support tool should not automatically be considered inappropriate, as no single document can guide all situations adequately, and we acknowledge the existence of alternate forms of guidance. There will always be exceptions to every “rule.” We intend to review and potentially revise the online components of each pathway as emerging data dictates, likely every 6-12 months, with significant changes and updates to be submitted for publication in the future as appropriate.

General recommendations: pretreatment management

Our panel recommends that, whenever possible, patients with oligometastatic disease be managed by an experienced multidisciplinary treatment team, including oncologic and supportive care providers. Critical considerations include the use of prognostic modeling to determine whether there is a likely benefit of “ablative”-type therapies, such as SBRT, vs. palliative approaches or supportive care. High-risk mutations such as TP53, ATM, BRCA1/2, or RB12 portend a potentially worse outcome, and for such patients, the scope of metastasis-directed therapy (MDT) and alternatives to radiotherapy should be considered [10]. Depending on the sites of metastases, whole-body (i.e., “eyes-to-thighs”) PET/CT with the optimal tracer is the preferred imaging option, with MRI or functional MRI as reasonable options when available. CT imaging is the minimum imaging needed. When SBRT is used, it should be performed with utmost precision to optimize efficacy and ensure patient safety. Quality assurance measures, such as phantom-based verification of treatment dose and delivery patterns prior to patient treatment, are essential.

General recommendations: treatment volume determination

The panel recommends expansion of the gross tumor volume (GTV) or internal target volume (ITV) to the planning target volume (PTV) be determined based on whether the target is the bone (with potential differences for vertebral bodies vs. other bones), soft tissue, or lymph nodes, as well as the capabilities of the radiotherapy equipment. In all situations, PTV coverage may be reasonably sacrificed (or the PTV margin reduced) to ensure sufficient protection of critical organs at risk (OAR). In general, bone GTV/ITV to PTV adjustment should be 5-10 mm. As noted above, such expansion may not be reasonable for vertebral bodies, and treatment in these cases may warrant special consideration, as discussed subsequently. Nodal target expansions may be reduced with accurate imaging (i.e., 2-10 mm). Such volumes may also include a clinical target volume (CTV) to address the concern for microscopic involvement of adjacent nodes, at the treating physician’s discretion and as anatomy allows. Furthermore, the expansions mentioned here should be modified when there is evidence of known setup certainty or uncertainty.

General recommendations: dose selection/prescription

The biologically equivalent dose (BED) and primary lesion or biopsied metastasis histology must be considered when selecting doses, with higher doses preferred for colorectal primary tumors, melanoma, and renal cell carcinoma [11,12]. There are two possible approaches to target dose volume assessment. The first approach is to use a homogeneous dose, wherein the PTV is covered by the 95%-100% isodose line (IDL). Alternatively, a heterogeneous dose distribution may be chosen, such that the PTV is covered by at least the 80% IDL, allowing for substantial internal hotspots. Consideration should be given to the evaluation of the conformity index, gradient index, heterogeneity index (HI), and inverse Paddick [13-15]. In the case of the Radiation Therapy Oncology Group (RTOG) conformity index, defined as the volume of the prescription isodose surface divided by the target volume, a value of 1.0 would be ideal, a value higher than 2.0 would violate protocol (at the time the parameter was established), and a value of 1.2 would be considered very good. The HI is defined as the ratio of the maximum dose in the treatment volume to the prescription dose, which indicates the magnitude of treatment plan “hotspots.” When established by the RTOG, a radiosurgery plan was deemed per-protocol if the HI was less than 2.0.

General recommendations: image guidance and motion management

Motion management is particularly important for targets in the chest and upper abdomen due to respiratory excursion. Optimally, the creation of an ITV should be done with a 4D CT by contouring individual phases of the scan. The combination of each phase of the GTV makes up the ITV, as defined by the International Commission of Radiation Units and Measurements (ICRU) [16]. Alternative means for generating an ITV include performing inspiration and expiration scans and calculating the dose using a free-breathing scan obtained at the same isocenter. Breath-hold techniques are acceptable for patients who can tolerate them and can obviate the need for creating an ITV. Alternative approaches include gated treatment delivery, in which the beam is activated only when the patient’s breathing cycle is within a stable range; tracking, in which the beam aperture or the linear accelerator itself moves with target motion; or direct tumor motion tracking, which is aided by internal or external fiducials or surrogates.

The motion of the tumor must be considered in any form of image guidance used. Typical methods include the use of cone-beam CT and cross-fire/orthogonal single-plane imaging. Implanted fiducial markers can aid in targeting when feasible. Advanced methods include 4D cone-beam CT imaging, which allows the radiation oncologist to observe tumor motion as the patient breathes. This is particularly useful for patients who cannot tolerate breath-hold techniques. For all targets impacted by respiratory excursion, abdominal compression can serve as an adjunct to limit such motion, provided it is reproducible.

For any type of image guidance, bony alignment is usually initially performed with refinement based on the position of the soft tissue target or ITV. For the treatment of multiple metastases with a single isocenter, such as lung nodules, it is critical to recognize the nodules may shift relative to each other and relative to bony anatomy. When metastases are separated by more than 5 cm, we recommend considering alignment and treatment delivery to each target individually.

General recommendations: concurrent systemic therapy

Concurrent systemic therapy and SBRT are topics of investigation; however, until more studies are available, we recommend that chemotherapy and other biologically and chemically targeted therapies be administered concurrently with patients treated with standard fractionation, but not during SBRT [17-19]. Concurrent immunotherapy with PD1/PDL1 inhibitors may be considered. The integration of systemic therapy and SBRT will be monitored and updated in future iterations of this pathway.

Site-specific recommendations: lung metastases

Our consensus for the treatment of lung metastases is summarized in Table 1. 

**Table 1:** General approaches to radiotherapy management of lung metastases MIP, maximum intensity projection.

	Gold	Silver	Bronze
Motion management	Motion inclusive ± gating (4D CT) tracking	Motion inclusive (normal inspiration and expiration, slow CT)	Free-breathing CT
Contouring	4D CT + MIP + PET fusion	MIP	Free-breathing CT
Peripheral tumors	10-12 Gy x 5; 15-18 Gy x 3; 12-12.5 Gy x 4	10 Gy x 5; 34 Gy x 1	Same
Central tumors	10-11 Gy x 5	12 Gy x 4; 7.5 Gy x 8	4 Gy x 12-15
Ultracentral tumors	7.5 Gy x 8; 6-7 Gy x 10	4 Gy x 12-15	2.5 Gy x 20; 2.0 Gy x 25-30
Nodal metastases	2.0 Gy x 25-30; 2.5 Gy x 20	No alternate recommendation	No alternate recommendation

The location of the tumor is critical for determining the optimal treatment approach, which may involve SBRT or more conventionally fractionated radiation therapy [20]. Peripheral tumors are defined as those located more than 2 cm from the proximal bronchial tree, with no PTV overlap with the mediastinum or pericardium. For such tumors, an SBRT dose of 48-60 Gy over 3-5 fractions is most commonly employed, though alternatives are available, as shown in Table 1. Central tumors are those located within 2 cm of the proximal bronchial tree and are most commonly treated with SBRT doses of 50-55 Gy in five fractions. Ultracentral tumors are those that overlap the proximal bronchial tree and/or critical mediastinal structures. For such patients, five-fraction (or shorter) SBRT regimens are not advised, though hypofractionated approaches, like those described in Table 1, are reasonable.

As with all forms of radiation therapy, consideration of normal tissue dose constraints is crucial. As with other sites of treatment, the HyTEC publications [21] and the compilations of Timmerman [22] provide the majority of dose limits recommended by the committee, as shown in Table 2.

**Table 2:** Normal tissue constraint recommendations for the treatment of lung metastases Values in this table are adapted from works by Grimm et al. [21] and Timmerman [22].

	Three fractions	Four fractions	Five fractions
Typical dose total	54 Gy (18 Gy/fx)	48 Gy (12 Gy/fx)	60 Gy (12 Gy/fx)
Chest wall (less PTV) limit	V40Gy < 5 mL, D_max_ 50 Gy, V30Gy < 30 cc	V43Gy < 5 mL, max 54 Gy, V30Gy < 30 cc	V45Gy < 5 mL, max 57 Gy, V30Gy < 30 cc
Total/bilateral lung limit	V20Gy < 10%, mean < 8 Gy	V20Gy < 10%, mean < 8 Gy	V20Gy < 10%, mean < 8 Gy
Brachial plexus limit	V22Gy < 3 mL, max point dose 26 Gy	V24.8Gy < 3 mL, max point dose 29.6 Gy	V27Gy < 3 mL, max point dose 32.5 Gy
Aorta limit	D_max_ 45 Gy, V39Gy < 10 mL	D_max_ 49 Gy, V43Gy < 10 mL	D_max_ 53 Gy, V47Gy < 10 mL
Trachea and large bronchus	D_max_ 43 Gy, V39Gy < 5 cc	D_max_ 47 Gy, V42.4Gy < 5 cc	D_max_ 50 Gy, V45Gy < 5 cc
Spinal cord	D_max_ 22.5 Gy, V15.9Gy < 0.35 cc	D_max_ 25.6 Gy, V18Gy < 0.35 cc	D_max_ 28 Gy, V22Gy < 0.35 cc
Stomach limit	D_max_ 30 Gy, V22.5Gy < 5 cc	D_max_ 33.2 Gy, V25Gy < 5 cc	D_max_ 35 Gy, V26.5Gy < 5 cc
Skin limit	D_max_ 33 Gy, V31Gy < 10 cc	D_max_ 36 Gy, V33.6Gy < 10 cc	D_max_ 38.5 Gy, V36.5Gy < 10 cc

The committee regards chest wall/rib constraints as a “soft” constraint, and the risk of pain or fracture should be weighed against the risk of inadequate tumor control.

Site-specific recommendations: hepatic metastases

The ACROPath® panel particularly recommends aggressive treatment of liver metastases in patients with oligometastatic colorectal cancer (and potentially other histologies) who have a limited number of metastases and are unable to undergo resection. In general, the committee regards patients with a limited number of metastases (i.e., 1-5 lesions), each measuring less than 5 cm in size, to be optimal candidates for liver SBRT. More important than the absolute number or size of the metastases is the amount of functioning healthy liver remaining after radiation treatment [23-26]. Treatment of hepatic lesions should be considered in any case of oligometastatic cancer with a limited number of unresectable metastases. It is also reasonable to consider treatment in those with oligoprogressive cancer, oligorecurrent cancer, or symptomatic polymetastatic cancer, especially when local control is a critical factor in the patient’s overall prognosis. Treatment of liver metastases should be approached cautiously and may be contraindicated in patients with more than oligometastatic disease or those with impaired hepatic function due to disease burden with hyperbilirubinemia (typically those with a Child-Pugh score of B9 or C) [27-29].

Consistent and reproducible patient positioning is critical and should include (as appropriate) abdominal compression, breath-hold techniques, respiratory gating, or the use of fiducial markers, particularly when tumor motion is greater than 5 mm. If tumor motion is less than 1 cm, an ITV may be sufficient; however, for motions greater than 1 cm, active motion management or amplitude-based gating is preferred.

Treatment of liver metastases can involve SBRT approaches, other hypofractionated treatment regimens, or, in carefully selected cases, whole-liver treatment. For SBRT, the dose range is broad, though the majority of treatments fall within 45-60 Gy in 3-5 fractions. Hypofractionated (non-SBRT) dosing is commonly administered over 15 fractions to a dose of 67.5 Gy and is appropriate for patients with acceptable liver function but a somewhat greater burden of disease [30]. It is recommended that at least 700 cc of normal liver tissue be spared (i.e., receives less than 15 Gy). For patients unable to tolerate more aggressive therapies, palliative whole-liver radiotherapy with a dose of 21 Gy delivered over seven fractions is reasonable [31].

Site-specific recommendations: brain metastases

Between 25% and 40% of cancer patients will develop brain metastases during the course of their disease, with cancers of the breast, lung, and melanoma being the most common primary sites. Early detection is critical to effective treatment and is typically achieved through high-resolution contrast-enhanced MRI of the brain. This imaging study should be part of the initial evaluation for small cell or neuroendocrine cancers and considered when symptoms warrant other malignancies. Double-dose gadolinium contrast may improve detection, particularly for small lesions. Optimally, the interval between imaging and treatment should be 10 days or less [32]. Over the past two decades, treatment strategies have evolved from combination radiosurgery and whole-brain radiation [33] to whole-brain radiotherapy with conformal avoidance of the hippocampi [34] and to stereotactic radiosurgery (SRS) or fractionated stereotactic radiotherapy (fSRT) alone.

**Figure 1:**
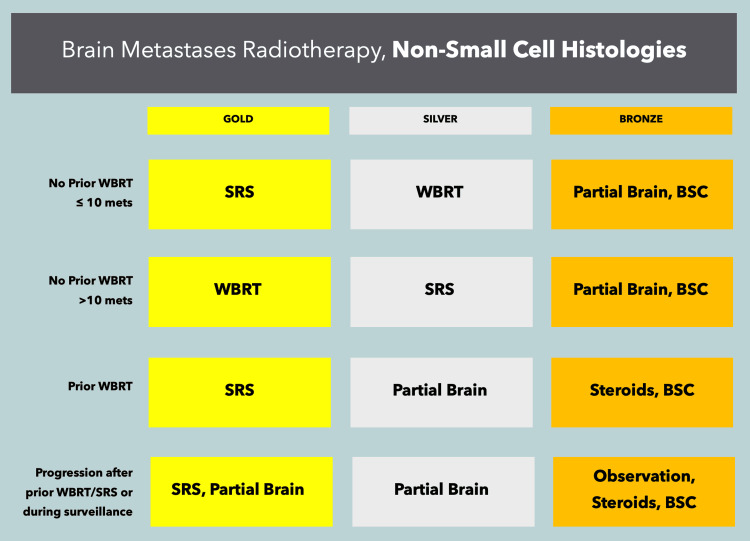
ACROPath® recommendations for the treatment of brain metastases from non-small cell malignancies SRS: stereotactic radiosurgery or fractionated stereotactic radiosurgery, WBRT: whole-brain radiotherapy, BSC: best supportive care, mets: metastases.

**Figure 2:**
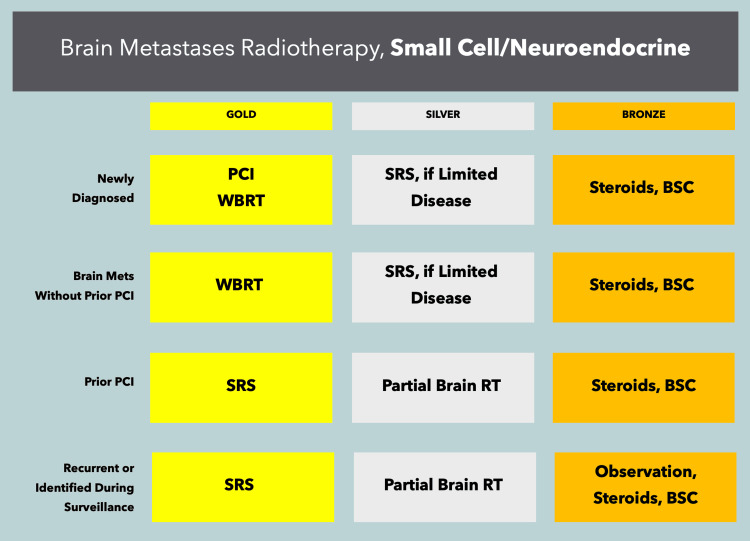
ACROPath® recommendations for the treatment of brain metastases from small cell and neuroendocrine malignancies PCI: prophylactic cranial irradiation, WBRT, whole-brain radiotherapy, SRS, stereotactic radiosurgery or fractionated stereotactic radiosurgery, RT: radiation therapy, BSC: best supportive care. For clarity, PCI regimens should not be used for patients who have radiographic evidence of existing metastatic brain disease, but rather as a preventative strategy in patients who are at risk of developing brain metastases.

In oligometastatic patients, SRS/fSRT alone is reasonable for tumors of any histology, provided there are a limited number of metastases in the brain. While the specifics of this approach are rapidly changing, the current maximum for this strategy is 10 brain metastases [35], though factors such as the treating radiation oncologist’s experience with multi-target approaches, geometric characteristics of lesions and their positions, and dosimetric limitations should be taken into consideration when determining any particular "limit." Surgery may be considered in highly selected patients who have symptomatic disease that is amenable to complete resection and controlled, limited, and/or no evident extracranial disease. Surgery may be particularly useful in patients with severe symptoms and/or significant edema. Whole-brain radiotherapy should be considered postoperatively in patients with extensive intracranial disease, those with more than 10 metastases, those with uncontrolled extracranial disease, and those with tumors of small cell or neuroendocrine histology. When used, whole-brain radiotherapy should employ hippocampal avoidance (HA), provided lesions are not within 5 mm of the hippocampi, or the avoidance structure should be modified accordingly [36]. Memantine is routinely used in whole-brain radiotherapy patients to reduce the neurocognitive impact of such therapy [37]. The gold/silver/bronze recommendations for approaching brain metastases are provided in Figure 1 for non-small cell histologies and in Figure 2 for small cell histologies.

For patients treated with whole-brain radiotherapy and HA, the committee recommends a dose of 30 Gy in 10-12 fractions, with dose limitations as specified by Gondi et al. [34]. For patients unsuitable for HA, a similar fractionation regimen may be used, though 35-37.5 Gy in 14-15 fractions is preferred by some. Doses for single-fraction radiotherapy typically depend on lesion size and histology, with lesions ≤ 2 cm receiving 21-24 Gy, those > 2 cm up to 3 cm receiving 18 Gy, and those >3 cm up to 4 cm receiving 15 Gy [38]. Fractionated SRS/fSRT dose data varies in the literature. Considering such data, the committee recommends that doses should be administered by size for three-fraction regimens, with those in the 2-3 cm range receiving 24 Gy and those in the 3-4 cm range receiving 27 Gy [39-41]. The dose for five-fraction fSRT is typically 35 Gy [42-44], and staged SRS for tumors ranging from 2-4 cm is administered as 18 Gy in one fraction, followed by a second fraction of 15 Gy one month thereafter [45]. Interested readers are again referred to the HyTEC papers and the Timmerman tables [21,22] for appropriate dose constraints. Treatment plan quality can be evaluated using metrics including conformity index, gradient index, HI, and inverse Paddick, as described previously.

Site-specific recommendations: bone metastases (non-spine)

The treatment of oligometastatic bone disease is indicated for symptomatic osseous lesions when the objective is palliation of bone pain and delay or prevention of disease progression, and it is indicated in asymptomatic osseous lesions when the goal is local control, delay of disease progression, postponement of further treatment, reduction of the risk of impending pathologic fracture, and/or to aid systemic treatment as part of a broader therapeutic strategy. Treatment should proceed with caution and could be contraindicated when blood counts are critically low, as further bone marrow exposure could exacerbate blood count deficits. Caution is also advised when using SBRT for large and expansile bone masses where doses could exceed normal tissue constraints.

Extrapolating from spinal treatment, higher doses of radiation have generally been associated with superior local control over traditional palliative regimens. Therefore, SBRT is appropriate for well-selected patients; however, traditional means may be preferred, depending upon treatment locations and limits imposed by adjacent OAR [46-48]. Available data on whether SBRT improves pain response over traditional means is conflicting; nonetheless, as part of an oligometastatic treatment strategy, we consider SBRT appropriate for local control and delayed progression. If SBRT is employed, particular attention to immobilization of the area treated is critical. Custom patient positioning devices are generally required, and for rib lesions, respiratory excursion must be considered, corrected for, and limited.

For treatment of the axial and appendicular skeleton (i.e., bone metastases other than those in the spine), five-fraction regimens typically deliver doses ranging from 7 to 12 Gy, depending on the size of the treated volume and proximity to the adjacent OAR. Other reasonable fractionation options include 48 Gy administered over four fractions and 27-36 Gy administered over three fractions. Single fractions of 16-24 Gy may be considered, depending on the volume and adjacent OAR. Normal tissue dose constraints typically dictate the optimal treatment fractionation. Overall guidance is provided in Table 3.

**Table 3:** General management approach to SBRT for non-spine bone metastases SBRT: stereotactic body radiation therapy, MDT: metastasis-directed therapy, BED: biologically equivalent dose, RTP: radiotherapy planning, Dx: diagnostic.

	Gold	Silver	Bronze
Indications for SBRT	Oligometastases, 1-5 sites	Oligoprogression or palliation/local control (e.g., spine)	Oligometastases, 5-10 sites (clinical trial or very select use)
Histology for MDT	Prostate cancer, lung cancer	Other histologies (e.g., breast, colorectal, sarcoma, renal cell, melanoma)	Breast cancer (very select use in oligoprogressive or conventionally staged only oligomet cases), small cell
Volume delineation/image fusions	RTP scan (soft tissue and bone windows), Dx CT scan, PET if applicable, MRI +/- C, 4D CT + MIP (ribs)	RTP scan Dx CT scan PET if applicable	RTP scan only three-phase respiratory RTPs (ribs)
Dose (BED3 Gy)	8 Gy x 5 (147); 10 Gy x 3 (130); 20-24 Gy x1 (153-216)	7, 9-10 Gy x 5 (117, 180-217); 12 Gy x 4 (240); 11-12 Gy x 3 (154-180)	9 Gy x 3 (108); 12 Gy x 2 (120); 16-18 Gy x 1 (101-126)

Site-specific recommendations: vertebral body/spine metastases

Unique among bone metastases are those impacting the vertebral bodies, owing to their immediate proximity to the spinal cord, cauda equina, and nerve roots. Vertebral bodies are also structurally essential weight-bearing bones, making it important to remember that vertebral fractures are a relatively common late side effect of high-dose radiotherapy [49]. SBRT offers promising potential for control, but alternatives should be considered, particularly when three or more contiguous vertebral bodies are involved. Acceptable dose ranges include 16-24 Gy in a single fraction, 24-30 Gy in three fractions, and 35 Gy in five fractions, with fractionated treatments delivered every other day. The risk of compression fracture increases with higher biologically equivalent doses. Sharp dose gradients are essential, particularly at the junction of the vertebral body and the thecal sac, with dose limitations outlined in the previously referenced works [21].

Site-specific recommendations: adrenal metastases

Though commonly observed as a site of metastasis, treatment of adrenal lesions poses unique challenges, owing to the proximity of the small bowel and the need to limit its dose. Optimal simulation is performed with oral contrast media administered 15-30 minutes prior to ensure adequate small bowel visualization. If SBRT is performed on bilateral nodules, there is a potential for lesions to shift relative to each other and bony metastases, so alignment to each side individually is typically required. If adequate motion management is not possible, more conventionally fractionated treatment may be required. The recommended approach to adrenal metastases is detailed in Table 4.

**Table 4:** ACROPath® treatment algorithm for adrenal gland metastases MIP: maximum intensity projection, ITV: internal target volume. For optimal simulation, administer oral contrast media 15-30 minutes prior for small bowel visualization.

	Gold	Silver	Bronze
Motion management	Motion inclusive ± gating (4D CT) tracking	Motion inclusive (normal inspiration and expiration, slow CT)	Free-breathing CT
Target definition	4D CT + MIP + PET fusion	MIP/ITV	Free-breathing CT
Size ≤ 5 cm and can meet 5-fx GI tract constraints	8-10 Gy x 5	5-6 Gy x 10; 7.5 Gy x 8; 6-7 Gy x 5	2.5 Gy x 20; 2.0 Gy x 25-30
Size > 5 cm or cannot meet 5-fx GI tract constraints	7.5 Gy x 8; 5-6 Gy x 10	4 Gy x 12	2.5 Gy x 20; 2.0 Gy x 25-30

Site-specific recommendations: lymph node metastases

SBRT as metastasis-directed treatment (MDT) for lymph node disease is effective, offering a high potential for local control and improved PFS with an overall low risk of severe toxicity when planned appropriately. When considering whether or not the disease is oligometastatic, immediately adjacent nodes are generally considered as a single site, and for thoracic disease, adjacent mediastinal and hilar nodes are considered a single site. Motion management should be considered for all areas impacted by respiratory excursion. In general, the CTV/ITV should be expanded by 0.8-1.5 cm for non-SBRT approaches and as feasible for SBRT approaches. Particularly with prostate cancer, treatment of the adjacent nodal bed should be considered. The strongest support for ablative treatment of oligometastatic nodal disease exists for prostate cancer, for non-small cell lung cancer (NSCLC), and for recurrent cancers of the head and neck. Supportive data also exists for colorectal cancer metastases, as well as for cancers of the breast, upper GI tract, gynecologic structures, and anus, in addition to melanoma and renal cell carcinoma [50-54].

The dose and treatment regimen (i.e., standard fractionation, hypofractionation, or SBRT) are based largely on the site of the nodal metastases. In the mediastinum, hypofractionated treatment of 45 Gy in 15 fractions is reasonable, as is SBRT using doses of 25-40 Gy in five fractions, provided OAR constraints can be met. For nodes in the abdomen, 24-30 Gy in three fractions or 33-45 Gy in five fractions may be feasible. In the pelvis, common regimens include 20-50 Gy in 3-5 fractions for SBRT. Standard fractionation is typically 50 Gy in 25-28 treatments, and hypofractionation with a simultaneous integrated boost (SIB) may be useful for the treatment of involved nodal disease and adjacent areas simultaneously. For SIB, the panel recommends consideration of 25 fractions using 1.8 Gy for the elective areas and 2.2-2.7 Gy for known diseases.

Origin-specific recommendations: prostate cancer

The data for which patients benefit most from MDT continues to evolve, and biomarkers for prognostic use are in development. For now, existing data suggests lymphatic and bone metastases from prostate cancer respond more favorably than visceral lesions, given the more aggressive and mutated state of prostate visceral metastases [55].

The SABR-COMET study provided encouraging data supporting aggressive treatment of oligometastatic disease with MDT vs. systemic treatment alone. In this phase 2 randomized controlled trial (RCT), 16% of those enrolled were prostate cancer patients. The 8-year OS for all patients nearly doubled from 14% with systemic treatment alone to 27% with the addition of MDT (p = 0.008) [56]. Also encouraging are the results from the STOMP trial, which compared MDT to surveillance in PET-detected recurrent prostate cancer [52]. The authors found a 34% androgen blockade-free survival in MDT patients vs. 8% in surveillance patients. Finally, the addition of SBRT to androgen blockade was demonstrated beneficial in both the EXTEND and ARTO trials, with the ARTO trial showing an improvement in biochemical failure-free survival and PFS [57,58]. The use of prostate-specific membrane antigen (PSMA) PET imaging has greatly improved our ability to identify oligometastases in prostate cancer and should routinely be used in evaluating patients with potentially recurrent disease.

The integration of SBRT and radioisotope therapy in the treatment of metastatic prostate cancer represents a promising advancement. It leverages the precision of SBRT to deliver high doses of radiation directly to tumors while using radiopharmaceutical agents to enhance the targeting and effectiveness of the treatment. This combined approach aims to improve survival rates and QoL for patients with metastatic prostate cancer.

A detailed review of completed critical trials [59], as well as ongoing clinical trials, suggests each lesion may be treated with one, three, or five fractions, depending on the local practice of specific institutions and treating physicians. We recommend incorporating next-generation imaging to determine GTV target volumes, recognizing that PET uptake does not correlate exactly with disease extent. In some cases, high SUV “shine” can overestimate tumor volume. Unique to prostate cancer as a disease site is that most clinical trials to date have utilized conventional imaging in the determination of the total number of sites of disease, as newer techniques such as PSMA PET were not yet available [60]. The dose is commonly prescribed to the minimum IDL that covers the PTV. Acceptable fractionations and dose levels suggested are as follows: for one fraction, the preferred dose is 20 Gy, with a range of 16-24 Gy; for three fractions, the preferred dose is 30 Gy, with a range of 24-33 Gy; for five fractions, the preferred dose is 35 Gy, with a range of 25-40 Gy. For targets adjacent to the kidneys, we recommend using SBRT delivered in 3-5 fractions, accounting for potential absorbed doses from specific radiopharmaceuticals.

In all cases, the actual dose and fractionation will depend on the size and location of the lesion and the surrounding normal tissue constraints, but the dose should remain within acceptable dose levels. In addition to other limits noted, OAR doses for surrounding normal tissue constraints can be in accordance with the UK 2022 Consensus on Normal Tissue Dose-Volume Constraints for Oligometastatic, Primary Lung and Hepatocellular Carcinoma Stereotactic Ablative Radiotherapy [61]. Note these are suggested constraints and are to be used as guidance only. Final radiotherapy plan evaluation must always remain the treating clinician’s responsibility.

Origin-specific recommendations: breast cancer

In general, oligometastatic breast cancer patients treated with SBRT have favorable outcomes compared to those treated with traditional palliative or supportive management, with studies demonstrating high control and low toxicity. Those with bone metastases tend to do better than those with non-bone metastases [62,63]. The previously cited SABR-COMET study population included 18% breast cancer patients, with nearly double the number surviving 8 years. The range of biological behaviors among breast cancers has made this patient subgroup challenging in some analyses, even in cooperative group trials [64]. The authors believe future data will further guide and refine the approach to SBRT in more well-defined subgroups of metastatic breast cancer patients.

Origin-specific recommendations: lung cancer

Patients with oligometastatic lung cancer are a particularly appropriate target for MDT due to the high incidence of oligometastases, which is reported to be 20%-50%. NSCLC patients with 1-5 metastases and an ECOG performance score of 0-2 are ideal candidates for aggressive treatment with SBRT. Iyengar et al. reported a trial in which PFS significantly improved from 3.5 months with maintenance chemotherapy alone to 9.7 months with the addition of SBRT (p = 0.01), with no in-field failures and fewer overall recurrences in the SBRT arm [51]. The CURB trial was a phase 2 RCT comparing SBRT to SOC palliative treatment in oligometastatic breast and lung cancer patients. The PFS in NSCLC patients was dramatic at 44 weeks with SBRT vs. 9 weeks with SOC (p = 0.004) [65]. Committee members regard the consideration of MDT in such NSCLC patients as critical to optimal care.

Origin-specific recommendations: other histologies

For less frequently occurring malignancies, SBRT appears effective, though less supporting data is available. We are encouraged by studies suggesting that a fairly low BED may be effective in the treatment of ovarian cancer metastases with SBRT, with efficacy seen in doses as low as 25 Gy in five fractions [66]. In metastatic melanoma, there are indications that coordinated treatment with SBRT combined with immunotherapy using nivolumab and ipilimumab improves OS in both symptomatic and asymptomatic brain metastasis patients [67]. The breadth of malignancies included in the largest oligometastatic disease trials leaves us to conclude that the merits of MDT likely extend to most malignancies.

## Discussion

The following represents the committee’s view on the optimal approach to managing oligometastatic disease for the sites and conditions described at the time of publication. While not all unique scenarios can be anticipated, we emphasize the importance of sound clinical judgment over strict adherence to even the most well-designed clinical support tools. As with all forms of medical care, we are mindful of the continuous evolution of oligometastatic disease management and encourage readers to stay informed of the significant updates as they are published. With that in mind, the committee eagerly awaits new evidence from clinical trials and patient outcomes to further refine this pathway.

As of this writing, ClinicalTrials.gov lists 50 phase 2 or 3 trials actively recruiting oligometastatic patients for radiotherapy interventions. These studies are available to patients across the USA through NCI’s cooperative group trial networks, including studies for patients with prostate cancer (NRG PROMETHEAN NCT05053152 [68], ECOG-ACRIN INDICATE NCT04423211) [69], head and neck cancers (ECOG -ACRIN NCT05721755) [70], esophageal and gastric cancers (ECOG-ACRIN NCT04248452) [71], and renal carcinoma (NRG SAMURAI NCT05327686 and ECOG-ACRIN SOAR NCT05863351) [72]. We encourage groups to consider opening these trials and enrolling patients through the NCTN or NCORP programs. In addition, many academic centers and pharmaceutical companies are testing the role of radiotherapy in combination with or as an alternative to systemic therapies. Together, these studies will provide key evidence to further develop the role of radiotherapy in the treatment of oligometastatic cancer.

The recognition of oligometastatic disease as a unique stage of cancer progression represents a critical change in our understanding of cancer biology. Broad application of its treatment may have a significant impact on longevity and QoL for patients with metastases. While the possibilities are promising, our panel advocates for the cautious and judicious use of aggressive therapies, as described above. We believe that the clinical pathways suggested herein will assist radiation oncologists in applying these techniques in an optimal manner.

## Conclusions

ACROPath® Oligometastases is the first in a series of clinical pathways in radiation oncology developed by ACRO. We believe this pathway offers a unique and detailed approach to optimal patient management. Along with upcoming pathways in lung, prostate, breast, head and neck, and rectal cancers, it offers clinicians a strategy to ensure patients receive high-quality, standardized care across centers in both the USA and internationally.
